# The Cognition of Severe Moral Failure: A Novel Approach to the Perception of Evil

**DOI:** 10.3389/fpsyg.2018.00557

**Published:** 2018-04-20

**Authors:** Aner Govrin

**Affiliations:** The Program for Hermenutics and Cultural Studies, Bar-Ilan University, Ramat Gan, Israel

**Keywords:** evil, moral development, prototype theory, moral judgments, cognitive bias, perspective taking

## Abstract

I describe the perception of evil as a categorization judgment, based on a prototype, with extensive feedback loops and top-down influences. Based on the attachment approach to moral judgment (Govrin, 2014, 2018), I suggest that the perception of evil consists of four salient features: Extreme asymmetry between victim and perpetrator; a specific perceived attitude of the perpetrator toward the victim's vulnerability; the observer's inability to understand the perpetrator's perspective; and insuperable differences between the observer and perpetrator's judgment following the incident which shake the observer no less than the event itself. I then show that the perception of evil involves a cognitive bias: The observer is almost always mistaken in his attributions of a certain state of mind to the perpetrator. The philosophical and evolutionary significance of this bias is discussed as well as suggestions for future testing of the prototype model of evil.

## Introduction

The term “evil” is often used to encourage an intolerant and extreme stance toward an enemy, or someone who violently opposes you. Over a period of thousands of years, the concept of evil was closely linked to a religious view of life. In Judaism and Christianity evil is viewed as human conduct in defiance of God's Commandments. An act of evil violates that holy code.

However, despite the evident religious connotations attached to the concept of evil, widespread usage of the term has survived. People in Western societies employ the term in a variety of contexts. The holocaust has become the ultimate paradigm of evil (Gampel, 2016, p. 1). However, the term is also used to describe war crimes, horrific acts of murder, cruel violence, sexual abuse and attempts to cause suffering simply to gain pleasure from a victim's acute distress. One must assume that the concept has survived because people find it useful. Perhaps it describes a category of moral failures of a certain kind better than any other concept does.

And yet, even though the term is quite common, psychologists have avoided using empirical methods to deal with the subject of evil. In the professional discourse, evil has been consistently viewed at best as an elusive topic and, at worst, a dangerous one and thus should not be, nor need it be, turned into a scientifically researched field of enquiry (Govrin, 2016).

This paper deals with the following question: What is it that glues together disparate acts of evil? In other words: Can we point to certain characteristics which are common to all instances of perceived evil? And if so can we say, as I shall argue that we can, that this commonality constitutes a prototype of evil? It is important to point out that the fact that one person terms a certain moral failure as “evil” and another disagrees, doesn't constitute a problem from a perceptual point of view. The two people concerned can agree on what the common features of all instances of evil are, even if they disagree about the existence or otherwise of these characteristics in a given moral situation. Therefore, cultural differences in relation to moral values do not necessarily cancel out the agreement that exists between people in relation to the perception of evil.

There are three parts to this paper. In part one, I show that it makes a great deal of sense to perceive evil as a coherent concept incorporating a number of salient features which, combined, create a prototype of evil.

In part two, I rely on the attachment approach to moral psychology (Govrin, 2014, 2018) to show that four co-occurring features generate the most salient features of the prototype of evil: Extreme asymmetry between victim and perpetrator; a specific perceived attitude of the perpetrator toward the victim's vulnerability (Govrin, 2016); the observer's inability to recognize or identify with the perpetrator's motivation; and insuperable differences between the observer's and perpetrator's judgment following the incident. In the third part, I describe the perception of evil as a cognitive bias, an inherent gap between the attribution of the observer and the actual experience of the perpetrator. The philosophical and cultural significance of this gap is discussed in the final part of the paper.

My objective is to inspire a greater interest among psychologists in the concept of evil, to view it as an important subject for scientific research and to stimulate an understanding of this phenomenon from a variety of perspectives. As a consequence of the paucity of research on this topic in the field of moral psychology I draw on evidence outside of that domain including evidence provided by historical events. Therefore, the model I am suggesting can only be regard as a possible explanation of what constitutes an act of evil and not as an established fact.

## Evil as prototype

Elsewhere I explained why philosophers had, and continue to have, a tough time defining evil (Govrin, 2016). Definitions of evil are plagued by three problems. Firstly, they are circular and employ formulations that describe evil's emotional impact (often in the shape of adjectives like “shocking,” “outrageous”) but not its essence; for example, according to Eve Garrard, “evil acts are not just very bad or wrongful acts, but rather ones possessing some especially horrific quality” (Garrard, 2002, p. 321).

Secondly, many of the philosophical definitions of evil are quantitative in the sense that they distinguish evil merely in terms of excessive wrongdoing.

Evil is portrayed as “very very bad” without pointing to the array of circumstances and conditions that are involved in the experience of evil. In these definitions, evil is described as something extreme by means of a metaphoric “graveness.”

As Calder (2012) argues, “If evil is just very wrong we can do without the term ‘evil.’ We can say everything we need to say using terms such as ‘very wrong’ or ‘very very wrong’ ” (178).

Thirdly, other definitions are partial and do not capture the gestalt of the concept.

Luke Russell (2007), maintains that no philosopher has been able to creditably depict an act of evil that is qualitatively distinguishable from commonly encountered acts of wrongdoing.

### Why is it so hard to tell what makes people judge an act as “evil”?

The difficulty with formulating a definition of evil is that most definitions rely on classical philosophical structures. The definitions I have cited try to isolate and apply an appropriate law or rule. They attempt to locate a set of necessary and sufficient conditions that would effectively define evil.

Schein and Gray (2014), call this kind of model an “if-then” paradigm. Such theoretical accounts, they explain, view the mind as if it were a highly skillful machine able to effortlessly calculate acts of evil. The calculation is made by subjecting a given situation to a series of simple tests with an example being classified as evil only if it passes all the tests in turn. Let us suppose that in order for an act to be classified as evil it must be massively damaging, intentional, and absent of any expression of remorse by the perpetrator. In the event that, for argument's sake, the perpetrator fails to express regret, then according to the “if then” criteria the act will be deemed “evil.” As Schein and Gray (2014) argue, such models were advanced decades ago by Alan Turing. Since then contemporary research has shown that in reality mental processes are far more complex (Dreyfus, 1981, 2007).

The if-then model represents a classical structure by which an appropriate law or rule is isolated. The trouble is that most human concepts do not possess a classical structure. Marc Johnson calls such a doctrine “moral law folk theory” (Johnson, 1993, p. 4). This doctrine, he claims, permeates our cultural heritage and hence underpins both lay and philosophical conceptions of moral life. Yet it is a doctrine that, he argues, is radically mistaken and morally incorrect. According to Johnson it would be ethically reckless for us to believe and behave as if we had within us a universal, ethereal faculty for reasoning which is capable of generating universally accepted laws and procedures (5).

Instead of static definitions, recent research suggests that moral concepts proceed “like a swirling vortex, pulling together cognitive elements toward an underlying prototype” (Schein and Gray, 2014, p. 236). Recent views of the operation of the mind support this more disorderly and more dynamic view of how judgments are formed.

In work carried out by Spivey and Dale (2004), perception and cognition have been shown to involve continuous processes of competition, rather than successive computations. Examples showing this to be so are drawn from extensive research in visual cognition.

A better account to define moral judgment is through a class of computational approaches known as connectionism. According to this view, we do not simply test for the presence or absence of a neat list of defining features and judge the concept applicable or inapplicable accordingly. One of the more fruitful models of this approach is theory offered by Churchland (1989, p. 104) which draws in neuroscientific findings.

According to Churchland's moral network theory (Churchland, 1996) our moral knowledge is developed in a process similar to that by which we develop specific physical skills, by training the response of neuronal networks to sensory input. Such training enables us to understand and adapt to the social world in which we live. Churchland maintains that as we acquire moral knowledge we learn to distinguish between morally “important” and morally “unimportant” categories of action and between what is morally “bad” and morally “good.” Moreover, Churchland unifies similar cases under one roof thus creating a core “hot spot” (Churchland, 1989) representing an archetypal example of that particular category.

Unlike Churchland (1996), Clark (1996, p. 113) argues that a prototypical model cannot be understood as an actual specific case. Instead, Clark suggests that the critical factor involved is the statistical median of a group of exemplars. Such a measure is computed by viewing each specific exemplar as consisting of several features that regularly appear together leading to the formation of a kind of artificial model which links the characteristics that are statistically the most significant. Thus, the archetypal pet may possess both dog and pet features, and the archetypical crime may include personal injury and loss of property. In Clark's view, specific models and “rich sophisticated know-how” remain key factors, but their role is to provide information on the basis of which these simulated models are formed. New cases then fall under the umbrella of a specific category (such as “pet” or “crime”) depending on how closely its features conform to those of the simulated model. Clark (1996) writes: “Features common to several training examples will figure in more episodes of weight adjustment than the less common features. As a result, the system will become especially adept at encoding and responding to such features. Feature that commonly occur together in the exemplars become strongly mutually associated. The system extracts the so-called central tendency of the body of exemplars, that is, a complex of common co-occurring features” (113).

Such a concept of prototype corresponds with a model of information storage in the brain called state-space representation, which draws on neuroscience (Churchland, 1989; Clark, 1996).

Churchland (1989) posits that the brain's representation of color, for example, is perceived as involving a three dimensional (3D) state space in which the dimensions reveal a long-wave reflectance, (b) medium-wave reflectance and (c) short-wave reflectance. According to Churchland, each such dimension may correspond to the action of three distinct types of retinal cone. Within such a 3D space white and black reside in diametrically opposed locations, while red and orange are quite close together. Our perceptions regarding the perceived similarity-difference relations between colors may thus be understood as mirroring distance in this color-state space.

According to Churchland, new instances are rather categorized as basically falling under a concept or category according to the perceived distance of the instance from a prototypical example.

Churchland's theory has been criticized by Larson (2017) on the grounds that it fails to identify which features of moral prototypes are crucial for categorization. Larson points out that Churchland is wrong to assume that comparable actions such as lying, cheating and betraying are a reliable way of categorizing “morally bad” behavior since those very same actions may be viewed in certain circumstances as “morally good.” For example, lying to a hostage taker to save the lives of the hostages. Such a lie is clearly in a different category than lies that are morally wrong. Larson posits that mere words such as “lying” or “cheating” do not capture the essential elements of moral failure.

Instead of thinking that perceivers of evil apply a rule-based context-free moral vision we must find what kind of fast, highly focused, context related information perceivers are considering when judging whether a moral failure is evil or just an act of severe wrongdoing. Although the categories of moral failure and evil overlap, evil tends to have greater weight and emotional response.

The tendency of philosophers to look for defining features should be replaced then by an inclination toward human moral psychology. We cannot understand evil without knowing a great deal about how the mind operates when facing moral situations, what crucial factors the mind weighs and how and what kind of interplay exists between motivations, emotions, and cognitions when making right/wrong judgments.

In this paper, I wish to base the perception of evil on a prototype model. As Schein and Gray (2014) have suggested, prototypical models forecast human cognition more accurately than do paradigms based on the “if-then models” (236) in every field of research in which the predictive capacity of these two models has been compared. I suggest that evil is no exception.

Burris and Rempel (2008), were the first to explore evil as a prototype. They initially asked approximately 200 students to list whatever came to mind when they thought of evil. Students' responses were coded into possible meaningful categories. Evil is perceived as applicable to events involving intentional harm, is associated with negative emotional reaction and with religious (Satan, Adam and Eve) and secular symbols (money, black). They posit that the that the term “evil” can be applied to acts viewed as coinciding with a prototypical model of harm, intent, and perceived lack of justification. People apply the label evil whenever enough of these central features of the evil prototype are salient in a given situation.

In my view, the traits singled out by this research are necessary but insufficient. Firstly, Gromet et al. (2016) found that an observer who takes pleasure in the suffering of others will be judged evil even if he was not responsible for the victim's suffering. Which is to say that even in the absence of “intentional harm” the behavior of those involved is categorized as “evil.” If so, classifying an act as “evil” must in some way be linked to the position thought to be held by the perpetrator toward the victim's suffering. Secondly, the characterization “perceived lack of justification” is insufficient. Severe negligence resulting in death or injury can also be judged as lacking justification but is not considered evil. Also, the perpetrator will have many justifications, considered by him to be valid, for having harmed the victim. Why, in so many cases, does the observer refuse to accept the perpetrator's explanations? Perceived lack of justification is too narrow a characterization to describe the huge cognitive discrepancy and emotional crisis between the observer and the perpetrator.

Thirdly, these characterizations do not consider the power relations between the two sides and the specific traits of each one of them.

Let us assume several cases:

A 7-year-old child shot another child for no apparent reason.

Or:

An incident in which an individual suffering from a psychiatric disorder shoots someone.

Or:

A case in which the murderer and the victim are both convicted criminals.

Each of these cases is characterized by intentionality, harm, and lack of justification. However, we cannot rule out the possibility that the observer will not classify these behaviors in the same way since in each case the relations between the two parties differ.

The proposition advanced in this paper is that in every moral judgment reached the observer must assess relations between two sides. The parameters relating to evil and every moral judgment cannot in and of themselves supply us with an all-inclusive list of the traits relevant to acts of evil and the perpetrator's motives unless they are combined with a theory explaining how the observer assess the relationship between two people.

The central argument I develop is that the perception of evil must be understood thorough acquaintance with the nature of moral judgment. Elsewhere (2015) I argued that evil is not only defined by the intention of the aggressor and his wickedness, or the magnitude of the harm caused. Each of these in isolation cannot serve our purpose. Rather, we need to find the perceptual properties that guide us in recognizing and discriminating evil from ordinary wrongdoing. Like the perception of color and sounds, this is not something we are necessarily aware of and here too we might find as in other cognitive faculties the priority of the preverbal over the verbal.

The perception model of evil presented here is a particular case within a general theory of moral judgment–the attachment approach to moral judgment (Govrin, 2014, 2018).

According to this theory, the core of most moral judgments is an observer evaluating a dyad. Thus, within a basic moral judgment situation three sides are involved: two conflicting parties (a dyad) and an observer.

**O Relates to the Following Dyad: A→C**

O-Observer
A Perceived wrongdoer.
C Perceived victim.
→ Behavior, Harm done, Overall attitude of A to C.

This theory emerges from a modest tradition of research according to which the foundation of morality is linked to our evolution as mammals that possess a system of attachment and an ability to feel and respond to the pain of others (Bowlby, 1958; Churchland, 2012; Haidt, 2012).

According to this theory, common to all moral situations is a universal deep structure which infants learn to identify rapidly and effortlessly in their first year of life. The deep structure behind every moral situation is a dyadic structure (Gray et al., 2012) between a side that is identified as a parental figure (the strong side) and a side that is identified as dependent and needy (the weak side). What activates this capacity is the interaction between the infant and the caregiver. By identifying relations between a dependent and the caregiver the infant acquires a range of expectations which are directed at the way in which a side identified as strong has to behave toward the side identified as weak. Moral judgment is a computational process whereby the observer calculates the child-like and adult-like features of each of the sides together with assessing the violation of expectations that may have occurred in the behavior of the strong side toward the weak side.

The perception of evil is based on the same parameters. I believe four salient features are found to be present at one and the same time when perceiving evil.

## Four salient features of evil

1. Asymmetry

Think of all the following dyads:

Rapist → Victim.

Nazi → Jew.

Child molester → 4 years old child.

There is one feature that is common to these crimes: An extreme perceived asymmetry between victim and perpetrator, the first salient feature required for the perception of evil. Whenever the observer identifies evil the victim or dependent is perceived as weak, helpless, defenseless, needy and, at times, innocent. The perpetrator, on the other hand, is perceived as strong and all-powerful: This type of extreme asymmetry may manifest itself through binaries like armed/ unarmed, adult/child, vulnerable/powerful, weak/strong, etc.

To constitute the dyad, the computing system takes both sides' features into account and checks their power relations. Any adult-associated characteristic, when somehow linked to the victim, is likely to moderate the asymmetry, and vice versa: Child-associated features attributed to the perpetrator will have the same unsettling effect. Think for instance about the subtle differences in each of the similar statements below:

The man pulled out his gun and shot the child in the head.

The man pulled out his gun and shot the mayor in the head.

The man pulled out his gun and shot the armed policeman in the head.

Or:

The 5 year old child pulled out his gun and shot the policeman in the head.

Or:

The 5 year old child pulled out his gun and shot the baby in the head.

Changes in the power relations that impact a situation are likely to affect moral judgment. The more obvious the difference in power, the more easily and faster is the judgment. These expectations are shaped by the parent-child dyad where one side is strong and has unlimited power, while the other is extremely vulnerable, weak and helpless. Where either side is attenuated by being ascribed one or more clashing features, expectations will change and moral judgment becomes harder to calculate.

But even here there might be gestalt shifts. When victims are “associated” with an evil actor, moral judgment will work against them and in favor of the perpetrator despite the asymmetry of power.

For example, suppose that we have the following information: thousands of innocent citizens were killed because of multitudes of aircraft bombings. Entire regions of the ancient beautiful city became hills of debris. At first, the asymmetry of power between the two parties (citizens of nation A, army of nation B) is obvious. Then we are told that it was the Allied Forces that sent the planes to bomb the German city of Dresden during WWII. The asymmetry of power is still present: The immensely powerful joint air forces of the Allies against innocent, helpless German citizens. However, the foreground becomes background: And so, the fact that they were citizens of Nazi Germany and the reality of that country's severe war crimes have a strongly moderating effect on people's moral judgment and make them consider the power conjunction quite differently. The information that the victims were Germans and that the bombing took place during WWII are not simply added on or incorporated into the original judgment. Rather, new meaning is given to the original judgment. What happened was a “component shift” (DesAutles, 1996, p. 135): A mental shift in how we perceive the dyad. This changes the computations of the different components and as a result the entire moral judgment.

Many other factors can moderate the asymmetry of force. For example, if the observer associates considerable personal distress with the aggressor—a distress that played a role in his reprehensible action—then this will weigh in in his favor. This then may well add vulnerable and needy features to the perpetrator and change the moral judgment, as for example, in the case of a husband killing his wife's lover. All this is also relevant to the next condition, namely the question of the accessibility of the perpetrator's state of mind to the observer.

2. The Perpetrator's Perceived Attitude to the Victim's Vulnerability

Another condition necessary for an observer to attribute evil relates to his perception of the perpetrator's attitude to the victim's dependency and vulnerability.

From the perspective of the observer, the perpetrator recognized the signs of extreme dependency displayed by the victim—helplessness, weakness—and nevertheless (and sometimes because of them) he knowingly and intentionally harmed him.

The observer's impression is that the perpetrator clearly recognized (as did he himself) a weak and helpless human being (or group). From the observer's perspective, the aggressor acted in full awareness of the victim's vulnerability.

But while in the observer this vulnerability and weakness arouse empathy and a desire to come to the victim's defense, the aggressor's perceived feelings are the very opposite. In some cases, the victim's vulnerability fails to arouse his concern, in others it even causes the aggressor to attack and injure him. Thus, the observer judges the perpetrator as hostile and aggressive toward the weak and needy. The aggressor's apparent awareness of the victim's neediness and vulnerability together with the suffering inflicted on the victim are what disturbs the observer and leads to the collapse of basic dyadic expectations, namely, that harming the dependent and weak is morally unacceptable and constitutes an act of evil. As Lazar (2017) writes on evil: “When we speak of catastrophe or collapse, we refer to an event that destabilizes thought and judgment, which does not allow presence and orientation” (202).

This is a qualitative, not a quantitative basis for attributing evil. It is the key condition distinguishing acts of evil from other grave moral failures. Thus, evil is not fundamentally in the act itself, nor in the gravity of the damage done, but is rather to be found in the perceived relation of the aggressor to the victim's vulnerability and weakness, and toward those who are needy and dependent in general. Bollas (1995) posit that “the evil person horrifies his victim and those who study him precisely because he lacks a logical emotional link to and is removed from his victim, even if transformed to fury” (189). Bollas's account mainly refers to serial killers in which “the evil one searches for someone who is in need and presents himself as good…when the victim takes up the offer of assistance, he becomes dependent on the provider; we may regard this form of dependence as malignant since the murderer feeds in order to destroy” (211).

In fact, as already mentioned, the two conditions to which I have so far referred—extreme asymmetry between perpetrator and victim, and the perpetrator's perceived attitude *vis a vis* vulnerability and neediness—are linked, the one influencing the other.

Between the two it seems that the latter is more informative and more salient.

As mentioned previously, Gromet et al. (2016) showed that people who derived pleasure from inflicting suffering on others, or were apathetic to that suffering, tend to be regarded by participants as immoral and evil whether their enjoyment was explicit or implied. That same judgment was also applied to people who merely observed a scene of suffering if they were perceived to have derived pleasure from the event.

At the same time, as Gromet et al's., research shows, there are circumstances in which the observer may derive a benefit from the suffering of the victim (for instance where the observer is promoted at the expense of the victim) but would not be judged as evil.

To illustrate how participants judge differing types of pleasure and their moral consequences, Gromet et al. (2016) describe a number of moral situations involving colleagues at work. In one situation a worker is seriously injured in an accident as a result of which he cannot for the time being return to work. The injured worker and a colleague are in competition for promotion. The accident removes the injured man from the competition and the promotion goes to his colleague—we could term this an “indirect pleasure.” In a second hypothetical situation, the two workers are not in competition and the uninjured worker doesn't gain anything from the other's misfortune other than “pleasure”—this could be said to yield “direct pleasure.” In a third situation the unharmed colleague has a mixture of emotions: pleasure at being promoted as well as sympathy for the victim and the injuries he sustained.

Only when direct pleasure was gained did the majority of people questioned, (75%), judge the colleague's response as evil. Participants had a clear preference to avoid physical or social contact with the actor who derived direct pleasure from his victim's distress. They expressed more comfortable feelings about being in the vicinity of the actor who gained “indirect” pleasure from such suffering and felt most comfortable about being associated with the person who reported mixed emotions.

The perceived stance of an individual toward the components of dependency and vulnerability in a victim appears to be the weightiest consideration in assessing acts of evil and in moral judgments in general.

All the observer's effort in any moral judgment is directed at assessing the aggressor's attitude to the victim's dependency-neediness component.

3. The Perpetrator's Mind Is Inaccessible to the Observer

The third salient feature required to match a prototype of evil involves the observer's shock and complete lack of understanding of the aggressor's motives. As Ronald Nasso (2016) writes: “We expect our lives to make sense, to fit within an intelligible framework. Evil challenges this cherished belief, confronting us with a world that is truly indifferent to our needs and wishes” (8).

The perpetrator's motives appear senseless to the observer. To the observer it seems that the perpetrator was either acting in an intentionally sadistic way, with a desire to harm, or displaying moral indifference. For the observer the perpetrator's act just does not make sense–he can't figure how the perpetrator does not see and perceive what he or she, the observer, sees and perceives. It is in precisely this sense to the observer the perpetrator's mind feels sealed.

As Lazar (2017) writes:” When we name an action evil [as opposed to crime], we actually mean that we do not know how to contain it within the existing order. Evil is an action which seriously threatens our trust in the world, a trust which we require in order to orientate ourselves within this world. Evil is characterized as that “thing” which massively attacks and collapses fundamental values cherished by man and society. Evil shakes the foundation, unraveling the important moral-emotional-relational tapestry of life, confusing any effort to build a cohesive explanatory scheme” (XIX–XX).

Even if the observer finds reasonable psychological motives to explain the aggressor's behavior the sense of bafflement remains. Often in such cases, the motives remain somehow external in the sense that they do not resolve the mystery surrounding the transgressor's actions. The observer understands but at the same time doesn't understand, is aware of the motives but remains guarded and unconvinced. For example, in *The Roots of Evil: The Origins of Genocide and other Group Violence*, Staub (1989) emphasized the predisposing societal conditions for genocide. Arguably, harsh living conditions as well as cultural factors may trigger certain psychological processes and provide motives that cause one group of people to assault another group thus launching a series of attacks which culminate in genocide (p. X). In situations of economic hardship and rapid social change people become more motivated to defend themselves physically and psychologically. They are more likely to engage in destructive acts if: Groups share a sense of both superiority and insecurity; have a history of devaluing others and aggressive behavior; are more oriented to obey authority; their culture is monolithic rather than pluralistic. Eager to regain a sense of comprehension of the world and their legitimate place in it, they are more susceptible to genocidal ideologies, particularly when promulgated by authoritarian governments which have the power to propagate a uniform definition of reality.

Staub helps us to understand the murderers and very accurately describes the psychological dynamics underlying genocide.

However, explanation and understanding do not share the same meaning and there is much debate within philosophy and psychology regarding their differences. Two principal theories of categorization emerge from this research: The *theory-theory of mind* and the simulation *theory of mind* (Zahavi, 2010). The theory-theory argues that our understanding of others mainly engages detached intellectual processes, moving by inference from one belief to another. According to Zahavi, the simulation theory of mind does not accept the idea that our comprehension of the behavior of others is largely hypothetical and argues that our own minds serve as a model when attempting to understand the minds of others. We approach others as if we share their beliefs and desires thus assuming a resemblance between us (Zahavi, 2010). Some researchers believe the two models are not mutually exclusive. In any case, explanations like Staub's seem to match the principles of the theory-theory, but not those included in the simulation theory: We understand the moral failure without reference to our own selves and feelings. We are unable to perceive an analogy between how we think and act and this particular terrible deed (Gallagher, 2005; Zahavi, 2005; Gallagher and Zahavi, 2008). It seems that we engage in comparing our mind to those of the perpetrators and only on this basis we feel we can't understand it.

Goldman (2006) has argued that an essential condition for mindreading “is that the state ascribed to the target is ascribed as a result of the attributor's instantiating, undergoing, or experiencing, that very state” (Goldman and Sripada, 2005; p. 208). Indeed, on Goldman's account “an attributor arrives at a mental attribution by simulating, replicating or attempting to do so” (194).

This is exactly the process that is obstructed in perceptions of evil. The observer may feel frustrated and shocked because of the extreme lack of correspondence between them and both his own and the aggressor's inability to locate zones of mutual harmony. Because the observer is identified with the dyadic rules this lack of correspondence is interpreted by him as the perpetrator's intention to act against our basic values and its catastrophic ruin of our moral matrix.

It is consequently no coincidence that Goldman considers a more apt name for the entire process to be simulation-plus-projection (Goldman, 2006, p. 40). Why, according to Goldman, is this circuit through self-deemed necessary? I need to project what I know about my own mind into the mind of others, because the only mind I have any direct and non-inferential knowledge of is my own. I know my own mind, but your mind is not present or manifest or given to me in any straightforward sense.

The observer's huge frustration here is obvious. He normally has no problems in successfully setting the circuits of simulation and projection into motion: Where he encounters the type of moral failure he identifies as evil this reliable everyday mechanism becomes useless. He simply fails to understand another person through himself.

As Dilthey (2010) also emphasized, when attempting to comprehend the behavior of the other that person's psychological state is not our principal interest. Rather we are seeking to decipher the meaning of his conduct and the extent of its legitimacy given that we live in a world we share and of which we have a common understanding. Understanding as well as self-understanding depends on a public scope of symbols, expectations and practices. It is in this profound sense that we fail to understand the true mind of the aggressor who commits what we consider an act of evil.

4. The Aggressor's Refusal to Accept Responsibility for His Deeds

Where the perpetrator's attitude after the act lacks remorse and regret and when he refuses to accept responsibility for his deeds, it might lead to the formation of two incompatible positions within the observer and the perpetrator. After the act, the perpetrator will be afforded an opportunity to alter the dyadic computation: He can express sincere regret about what he has done, accept responsibility and alter his stance toward the victim.

The aggressor in some cases understands that he has seriously failed to meet the expectations of how a strong person conducts himself toward a weaker one. By consequence, he understands that he has wholly and offensively ignored the fact that he was in the role of the strong one and the victim was weak: That there is an extreme imbalance of power between the two of them. He learns to see the victim's pain for the first time, the suffering he has inflicted and the indifference or cruelty with which he treated the victim. In doing so he comes much closer to the observer's position. He might be appalled by his own actions just like the observer.

Sometimes this can have the effect of reassuring the observer. Remember, that from the observer's point of view, the most problematic aspect of the situation is the fact that the perpetrator's action manifestly violated the rules of the dyad, the most blatant violation being the aggressor's refusal to recognize dependency / vulnerability as worthy of protection. That not only turns the aggressor into a dangerous and inhumane person but also undermines the observer's world view: The perpetrator's actions have shattered what the observer considers to be obvious, certain and axiomatic to the understanding of human nature. If the aggressor expresses sincere regret and is prepared to pay a price for his misdeed, compatibility between observer and aggressor could be reinstated. While the transgression is still perceived as very serious and remains unforgivable, some aspect of the fundamental moral matrix within which the observer conducts his affairs is restored. The observer feels more at ease as the dyadic rules have triumphantly reemerged within the perpetrator's mind. The expression of regret may also affect another one of the four criteria for attributions of evil: It may reduce the perception of extreme asymmetry between the sides because, having expressed regret, the aggressor is now perceived as more humane and vulnerable. It is as if the aggressor has once more become part of the human community: The object he perceives is like that seen by the observer.

On the other hand, the perception of evil is reinforced if the aggressor refuses to alter his stance. It might be said that the aggressor's attitude to—or computation of—the relevant dyadic situation diverges crucially from that of the observer. For example, he might see himself as a victim or emphasize factors that were beyond his control.

Scully's (1990) interviews with convicted rapists yielded two categories. *Deniers* justified their actions because the victim was willing or got what they deserved. Rapists' claims that their victim seduced them or had a reputation of sleeping around are examples. Deniers did not think they really had committed rape and were said to be “unaware of their victim's feelings.” In contrast, *Admitters*, acknowledged they committed rape but excused their actions by denying responsibility and blamed alcohol or some personal problem they had for their behavior; some even claimed that rape itself had become an addiction.

Observers are likely to remain indifferent to such explanations. The explanations don't make it any easier for the observer to recognize or identify with the aggressor's position. At times they may have the opposite effect and reinforce the attribution of evil.

It is hard imagining any explanation that could cause the observer to feel more affinity with someone who raped and murdered his victim. Explanations, as mentioned, will only tend to underline how different the experience of the aggressor is from that of the observer. The aggressor too acts within the rules of the dyad. He has his own moral judgment as to what happened. For example, if he was under the influence of alcohol the implication is that he was less responsible for his actions which is to say that his adult like components were weakened. One can say that both deniers and admitters are responsible for significant moral failures: Deniers are responsible for not acknowledging the dyad as a whole: They do not seem to grasp the asymmetry of force between them and the victim, the vulnerability of the victim, the suffering, and the serious and inexcusable harm. Admitters are responsible for their refusal to recognize that what they did cannot be justified and is morally inexcusable. The perception of evil comes as a response to the second moral failure no less seriously than it does to the first.

And so, the observer finds himself emotionally shaken once more by the way the aggressor perceives his own moral failure.

In *Unspeakable Acts: Why Men Sexually Abuse Children*, Pryor (1999) tries making the minds of the men who sexually abused children more accessible to readers. He does so by preparing the reader for a reading experience that is hard to digest emotionally:

Some men cried when they described what they had done; others became extremely angry with themselves; still others shook their heads in disbelief at what they were saying. What I discovered was the human side of the men; I found that their life had often been filled with what to them was pain and turmoil, and that many, though not all, I believe, were genuinely sorry for the acts they had committed (10).

These interviews give us a glimpse into the mind of the pedophile criminal. From Pryor's book we learn that most of the men interviewed had been sexually abused as children. But in my view what makes their position more understandable to readers is rather the aggressor's expressed stance, with hindsight, *vis a vis* the crime he committed. That stance is fully compatible with the position adopted by the observer. The pedophiles who participated in this research express horror at their own actions, admit their moral failure. They seem to be in shock because of what they did, and this matches the observer's response. If there is an attitude that can alter the observer's moral computation in these cases—which is not at all certain—it can only be by the aggressor perceiving the dyad in much the same way as the observer perceives it with the same degree of horror and the same level of incredulity in the face of his blatant violation of expectations.

Bear in mind that as is the case with every moral judgment there is no question of expecting an objective assessment of the four perceptual traits of evil. Different judges will weigh the importance of the various traits differently.

There are also personality factors which influence the judgment of an act of evil. Webster and Saucier (2013; see also Campbell and Vollhardt, 2013; Webster and Saucier, 2015, 2017) developed an individual difference scale of Belief in Pure Evil (BPE) assessing the degree to which individuals attribute sadistic tendency to other people. Individuals who more strongly believe in pure evil (who score higher on the BPE scale) exhibit a more antisocial/aggressive orientation toward others. Such individuals believe that the world is a viler, more dangerous place and report more aggressive (vs. peaceful) attitudes, from matters of foreign policy to the criminal justice system. Two studies have shown that people who in general have a stronger belief in pure evil recommend harsher punishments for a variety of crimes (murder, assault, theft), support the death penalty more strongly, and are more vehemently opposed to criminal rehabilitation (Webster and Saucier, 2013).

## Evil as cognitive bias

Mills asks: “ Does evil exist, or is it a social invention?” (Mills, 2016, p. 19).

Are the observer's attributions of evil appropriate? Is the observer right to assume that the person the perpetrator sees before him is the same as the person he himself sees–a vulnerable and weak victim? Is it true that where the observer identifies evil, the aggressor violates the dyadic rules, and lacks the natural, human, instinct in the face of suffering and distress? My main argument is that the observer is wrong in making these attributions (Govrin, 2016). The perception and attribution of evil are forms of cognitive bias and they are not independent of human creation and invention.

Roy F. Baumeister's book *Evil: Inside Human Violence and Cruelty* (Baumeister, 1997), for the most part examines the way in which perpetrators understand actions of theirs which have been judged to be “evil.” As is not uncommon in other severe cases of criminality, those responsible for such actions frequently believe that their conduct was wholly or almost wholly justified in response to what they perceived as an act of aggression by their “victim.” In Baumeister's view, difficult as it may be for some observer's to swallow, such opinions often contain an element of truth. Most people's view of evil is straightforward: a cruel, violent aggressor attacking a helpless victim. But not all perceived acts of evil conform to this description. Violence between two sides is often the result of ever worsening relations for which both parties to the conflict bear a share of the responsibility. Thus, the side perceived by observers as the aggressor may, at least in part, be justified in claiming “provocation.” Basing himself on a host of recorded cases Baumeister argues that in reaching their judgment in such instances of perceived evil the observer tends to underestimate the influence of the aggressor's situational circumstances and to exaggerate the extent to which the perpetrator's perceived temperament was responsible for his actions.

Our perception of evil is not rational. It is an error. It is not based on logic. And yet when we perceive evil we think of it in absolute terms, and our perception as Roth explained “is not open for debate” (Roth, 2017, p. 182). However, like many other biases this is not a design flaw of our mind but a design feature (Haselton et al., 2016).

It supports the assumption that the perception of evil is domain-specific and was favored by natural selection over an accurate perception of the perpetrator. In terms of our own survival we are fortunate to err. If people would perceive evildoers in an “objective” way, taking the perpetrator's perspective into account, they would probably be in danger. More than anything, the perception of evil involves fear: It signals an existential threat. Humans have an inborn tendency to experience fear in the face of certain stimuli like, for instance, snakes, spiders, water, and closed spaces. These fears are not consciously controlled. They occur even when the same objects are not dangerous (a non-poisonous snake), and even when we have had no earlier experience with them.

Nesse (2001), argued for what he calls a “smoke detector” principle in bodily systems. He cites a number of examples related to medical conditions such as allergy and cough where a defensive system is often battle-ready, even though there is no real danger. These protecting systems appear to be over-responsive.

That said, the perception of evil is also not without its dangers. Behind the most horrific violence people have inflicted on each other there is a perception of evil. That is, a claim by the perpetrator that his victim is an enemy and that because of his evil deeds he deserved his fate. In the years before the Holocaust, for example, the Nazi campaign of incitement painted Jews as evil, dangerous, and an enemy of the nation, thus justifying their conduct. And so, the perception of evil which is so crucial in maintaining the stability and security of human society, is also responsible for humanity's worst crimes.

### Future testing of the evil prototype model

According to the model suggested in this paper, recognizing a moral failure as evil is limited to cases which include the following salient traits: An extreme asymmetry between the sides, an attribution of indifference to the victim's traits of dependency (such as suffering) by the perpetrator (who may even derive pleasure from abusing his victim), the observer's inability to understand the aggressor's perspective, and the absence of remorse on the part of the aggressor following the incident.

The model can predict several interesting aspects linked to the perception of evil. Firstly, the perception of evil does not necessarily have to be linked to the extent of physical or psychic harm suffered by the victim. For example, the theory will predict that if Jon were to place an obstacle in front of David who is blind and deaf, and Jon gets much pleasure when his classmates burst out laughing his action will be judged as evil even though David did not even notice that he was being abused. The incident fits in with the prototype of evil at least in so far as its first three features are concerned.

Secondly, the attitude attributed to the aggressor in relation to the victim's vulnerability during the act is more important than any other factor. Thus, for example, a soldier who fires at a child during war because he mistakenly thought that that the child was a terrorist cannot be compared to a soldier firing at a child deliberately to kill him. The outcome in both instances is identical–a dead child. However, only the second incident fits in with the prototype of evil because the soldier in fact recognized the victim's vulnerability and neediness from the outset but nonetheless shot and killed him.

Thirdly, this model of evil is sensitive to any change in the child/ adult traits of either the victim or the aggressor. Every detection of vulnerability, weakness, or neediness on the part of the aggressor can distance the moral failure from the prototype of evil. How, for example, would the judgment differ in the case in which the blind David stumbled on an obstacle deliberately placed in his path by Jon, if we were to learn that Jon is a rejected and abused child who was trying to draw the attention of classmates by tripping David up? This additional information may distance the incident from the evil prototype, or it may not. In brief, we can say that an alteration of the child/adult traits on either side will lead to a change in the perception of evil.

Fourthly, the prototype model of evil sheds new light on the influence that an aggressor's expression of remorse has on the observer. To distance himself from the attribution of evil the aggressor has to convince the observer that there is a match between their respective views of what had happened. In other words, the aggressor must unequivocally accept the observer's view in relation to such components of the incident as the estimate of the extent to which the victim was harmed, the extent of the aggressor's responsibility for that harm, and that there had been a flagrant breach of expectations by the aggressor. The aggressor also has to be shocked by what he had done and show that he has difficulty in understanding his motives. The prototypical model of evil predicts that only a close correlation between the observer's and aggressor's perceptions after the incident will be sufficient grounds for the observer to distance the penetrator from being labeled as evil.

## Conclusions

In this paper, I have suggested that the perception of evil relies on a prototype with a unique set of features. My central assumption is that psychological research of the perception of evil is important and that research into moral judgment must include the perception of evil. An understanding of the perception of evil may have profound implications for the understanding of psychological phenomena in both the past and present.

Thus, for example, two historians, Browning (1992) and Goldhagen (1996) have offered two different approaches to explain the participation of so many “ordinary” Germans in the murder of the Jewish People in the course of WWII.

Browning (1992) thought that the motive of ordinary Germans who took part in the genocide was above all obedience to authority and peer pressure. In contrast, according to Goldhagen (1996) German society was profoundly anti-Semitic prior to Adolph Hitler's election. The Nazis, he argues, became rulers of a country ready to be harnessed to the idea that Jews were an enemy of the German People. From that it was a short step for the regime to justify their total annihilation.

The subject became the focus of fierce debate.

The model of evil can explain why. Behind the debate between the two historians there is something more profound: Is there a similarity between the acts of ordinary Germans who took part in the extermination of the Jews and the prototype of evil?

If we apply the two historians' arguments to the model described here, we can say that Browning (1992) attempts to show that three of the perceptual traits included in the prototype of evil are missing in the behavior of “ordinary” Germans during the Nazi era. Firstly, there is no extreme asymmetry between victim and perpetrator given that the German people were themselves victims of the Nazis and their regime of terror and fear. Secondly, since “ordinary” Germans only participated in the horrific deeds because they were obeying authority and the group's ethos one shouldn't consider them to have been either indifferent or sadistic in their attitude toward their Jewish victims. Thirdly, since their participation in the horror was not prompted by hatred of Jews but rather by obedience to the Nazi orders, their mind became far more accessible to the observer. After all, Milgram (1963) in his famous experiment found that ordinary American people would administer an electric shock to a learner that gave a wrong answer. Two third of his participants continued to the highest level of 450 volts. The obvious conclusion invited by this line of argument is that the actions of “ordinary” Germans while the Nazis were in power should not be regarded as evil.

Of course, Browning never said that. After all, Germans who participated in the genocide showed no empathy whatsoever to the Jew's suffering and no regret, one of the most defining features of perceived evil. However, it does shake the polarity between the innocent victim and the murderous perpetrator because it shows that the role of ordinary Germans in the genocide was an accident and that almost anybody could have taken their place (see also Arendt's answer to Eichmann's defense, Arendt, 1963, p. 278). It also makes the perpetrators mind much more accessible.

If, on the other hand we take Goldhagen (1996) arguments and view them through the prism of the model of evil which I propose we would conclude that the perceptual traits included in the model are evident in the behavior of “ordinary” Germans during the Nazi period: Germans, Goldhagen argues, participated in the genocide out of their own free will without the Nazi regime forcing them to do so (As proof of this he cites the cases of the few who asked and were permitted to be excused from participating without any sanctions). The Germans who acted out of free will and choice have to be regarded as possessing the traits of an adult and not of a child when compared to their weak and helpless victims. From Goldhagen's argument it follows that there was extreme asymmetry between the sides. Secondly, their deep hatred of Jews, according to Goldhagen, is evident from the fact that despite being weak and helpless the Jews didn't lead to a desire on the part of “ordinary” Germans to protect them and abstain from committing brutal acts against them. In this they were adopting an inhumane view of the suffering endured by the victims. Thirdly, as Goldhagen would argue, since “ordinary” Germans were motivated by a deep hatred of Jews the observer would be unable to accept their perspective or identify with their motives.

If we think that the Holocaust was a lesson which has to be learned and that everyone who finds themselves in such extreme circumstances can act in evil ways, then Browning (1992) explanation is to be preferred. On the other hand, Goldhagen's relates to the Germans who participated in the genocide as a “separate species” and refuses to show an understanding of their motives. In doing so he displays a greater sense of horror toward the actions of the Germans than does Browning.

This amounts to a Gestalt-shift: With Browning we are looking at the understandable and human motives of ordinary Germans obeying the powerful authority, whereas Goldhagen's analysis exposes the suffering of the victims and the brutality directed at them.

The prototypical model of evil can be applied to many other instances. Evil may indeed be in the eyes of the beholder, but this paper suggests that judgments of evil are not arbitrary, nor do they arise from a neat list of defining features. Rather, evil is a case of prototype based reasoning and is judged quickly, effortlessly, and without deliberation. The model can assist us to break down the concept of evil into its various components parts and then reexamine the extent to which the deed matches the model. Our spontaneous judgment of evil might prove to be false, but only if we are consciously aware of how our automatic perceptual system reached such a judgment we can reconsider our verdict.

## Author contributions

The author confirms being the sole contributor of this work and approved it for publication.

### Conflict of interest statement

The author declares that the research was conducted in the absence of any commercial or financial relationships that could be construed as a potential conflict of interest.
